# Supplementary material for: Prevalence and determinants of occupational injuries among emergency medical technicians in Northern Ghana
Source: PLoS One. 2023 Apr 25;18(4):e0284943. doi: 10.1371/journal.pone.0284943 (PMC10129006; doi:10.1371/journal.pone.0284943)
Supplement: Attachment — Submitted filename: PONE-D-23-04075_reviewer-BJM.pdf [file pone.0284943.s003.pdf]

PLOS ONE

Occupational injuries among Emergency Medical Technicians: Burden and determinants in Northern Ghana

--Manuscript Draft--

**Manuscript Number:**	PONE-D-23-04075
**Article Type:**	Research Article
**Full Title:**	Occupational injuries among Emergency Medical Technicians: Burden and determinants in Northern Ghana
**Short Title:**	Injuries among Emergency Medical Technicians
**Corresponding Author:**	Douglas Aninng Opoku, MPH<br>Allen Clinic<br>Kumasi, Ashanti Region GHANA
**Keywords:**	Determinants of occupational injuries; Emergency Medical Technicians; Health and Safety measures; Ghana; Occupational injuries burden
**Abstract:**	Background: Emergency Medical Technicians (EMTs) are the main actors in the provision of prehospital emergency medical services. The operations of EMTs increase their risks of being exposed to occupational injuries. However, there is a paucity of data on the burden of occupational injuries among EMTs in sub-Saharan Africa. This study, therefore, sought to estimate the burden and determinants of occupational injuries among EMTs in the Northern part of Ghana.<br>Methods: A cross-sectional study was conducted among 154 randomly recruited EMTs in the Northern part of Ghana. A pre-tested structured questionnaire was used to collect data on participants' demographic characteristics, facility-related factors, personal protective equipment use, and occupational injury. Multivariate logistic regression analysis with a stepwise approach was used to examine the determinants of occupational injury among EMTs.<br>Results: Over a period of 12 months, the prevalence of occupational injuries among EMTs was 38.6% with an overall 12-month incidence rate of 386 injuries per 1000 workers. Bruises (51.8%), and sprains/strains (14.3%) were the major types of injuries reported among the EMTs. The key determinants of occupational injury among EMTs were an absence of a health and safety committee at the workplace (aOR: 3.92, 95%CI: 1.63 – 9.43), absence of health and safety policy at the workplace (aOR: 2.76, 95%CI: 1.26 – 6.04) and dissatisfaction with health and safety measures at the workplace (aOR: 2.51, 95%CI: 1.10 – 5.71). Female EMTs had a 70% (aOR:0.30, 95%CI: 0.12 – 0.71) protection of sustaining occupational injury compared to the male EMTs.<br>Conclusion: Approximately three out of ten EMTs reported an occupational injury within a twelve-month period. Potential strategies to reduce the incidence of occupational injuries include the establishment of health and safety committees, formulation of health and safety policies as well as improving current health and safety measures among EMTs.
**Order of Authors:**	Ali Baba Awini, MPH
	Douglas Aninng Opoku, MPH
	Nana Kwame Ayisi-Boateng, FGCPS
	Joseph Osarfo, PhD
	Alhassan Sulemana, PhD
	Isaac Kofi Yankson, PhD
	Maxwell Osei-Ampofo, FGCPS
	Ahmed Nuhu Zackaria, FGCPS
	Sam Newton, PhD
**Additional Information:**	

Powered by Editorial Manager® and ProduXion Manager® from Aries Systems Corporation

Question	Response
**Financial Disclosure**<br><br>Enter a financial disclosure statement that describes the sources of funding for the work included in this submission. Review the submission guidelines for detailed requirements. View published research articles from *PLOS ONE* for specific examples.<br><br>This statement is required for submission and **will appear in the published article** if the submission is accepted. Please make sure it is accurate.<br><br>**Unfunded studies**<br>Enter: *The author(s) received no specific funding for this work.*<br><br>**Funded studies**<br>Enter a statement with the following details:<br>• Initials of the authors who received each award<br>• Grant numbers awarded to each author<br>• The full name of each funder<br>• URL of each funder website<br>• Did the sponsors or funders play any role in the study design, data collection and analysis, decision to publish, or preparation of the manuscript?<br>• **NO** - Include this sentence at the end of your statement: *The funders had no role in study design, data collection and analysis, decision to publish, or preparation of the manuscript.*<br>• **YES** - Specify the role(s) played.<br><br>* typeset	The authors received no specific funding for this work
**Competing Interests**<br><br>Use the instructions below to enter a competing interest statement for this submission. On behalf of all authors, disclose any competing interests that could be perceived to bias this work—acknowledging all financial support and any other relevant financial or non-financial competing interests.	The authors have declared that no competing interests exist

This statement is required for submission and **will appear in the published article** if the submission is accepted. Please make sure it is accurate and that any funding sources listed in your Funding Information later in the submission form are also declared in your Financial Disclosure statement.<br><br>View published research articles from *PLOS ONE* for specific examples.<br><br>**NO authors have competing interests**<br><br>Enter: *The authors have declared that no competing interests exist.*<br><br>**Authors with competing interests**<br><br>Enter competing interest details beginning with this statement:<br><br>*I have read the journal's policy and the authors of this manuscript have the following competing interests: [insert competing interests here]*<br><br>* typeset	
**Ethics Statement**<br><br>Enter an ethics statement for this submission. This statement is required if the study involved:<br><br>• Human participants<br>• Human specimens or tissue<br>• Vertebrate animals or cephalopods<br>• Vertebrate embryos or tissues<br>• Field research<br><br>Write "N/A" if the submission does not require an ethics statement.<br><br>General guidance is provided below. Consult the submission guidelines for detailed instructions. **Make sure that all information entered here is included in the Methods section of the manuscript.**	Ethical approval was granted by the Committee on Human Research, Publications and Ethics, School of Medicine and Dentistry, Kwame Nkrumah University of Science and Technology, Kumasi, Ghana with reference number CHRPE/AP/403/20. Study participants were briefed about the study objectives, risks and benefits of participation. They were encouraged to ask questions for clarification. All study participants signed informed consent before recruitment.

**Format for specific study types**<br><br>**Human Subject Research (involving human participants and/or tissue)**<br>• Give the name of the institutional review board or ethics committee that approved the study<br>• Include the approval number and/or a statement indicating approval of this research<br>• Indicate the form of consent obtained (written/oral) or the reason that consent was not obtained (e.g. the data were analyzed anonymously)<br><br>**Animal Research (involving vertebrate animals, embryos or tissues)**<br>• Provide the name of the Institutional Animal Care and Use Committee (IACUC) or other relevant ethics board that reviewed the study protocol, and indicate whether they approved this research or granted a formal waiver of ethical approval<br>• Include an approval number if one was obtained<br>• If the study involved *non-human primates*, add *additional details* about animal welfare and steps taken to ameliorate suffering<br>• If anesthesia, euthanasia, or any kind of animal sacrifice is part of the study, include briefly which substances and/or methods were applied<br><br>**Field Research**<br><br>Include the following details if this study involves the collection of plant, animal, or other materials from a natural setting:<br>• Field permit number<br>• Name of the institution or relevant body that granted permission	
**Data Availability**<br><br>Authors are required to make all data underlying the findings described fully available, without restriction, and from the time of publication. PLOS allows rare exceptions to address legal and ethical concerns. See the PLOS Data Policy and FAQ for detailed information.	Yes - all data are fully available without restriction

Powered by Editorial Manager® and ProduXion Manager® from Aries Systems Corporation

A Data Availability Statement describing where the data can be found is required at submission. Your answers to this question constitute the Data Availability Statement and **will be published in the article**, if accepted.<br><br>**Important:** Stating 'data available on request from the author' is not sufficient. If your data are only available upon request, select 'No' for the first question and explain your exceptional situation in the text box.<br><br>Do the authors confirm that all data underlying the findings described in their manuscript are fully available without restriction?	
**Describe where the data may be found in full sentences. If you are copying our sample text, replace any instances of XXX with the appropriate details.**<br><br>• If the data are **held or will be held in a public repository**, include URLs, accession numbers or DOIs. If this information will only be available after acceptance, indicate this by ticking the box below. For example: *All XXX files are available from the XXX database (accession number(s) XXX, XXX.).*<br>• If the data are all contained **within the manuscript and/or Supporting Information files**, enter the following: *All relevant data are within the manuscript and its Supporting Information files.*<br>• If neither of these applies but you are able to provide **details of access elsewhere**, with or without limitations, please do so. For example:<br><br>*Data cannot be shared publicly because of [XXX]. Data are available from the XXX Institutional Data Access / Ethics Committee (contact via XXX) for researchers who meet the criteria for access to confidential data.*<br><br>*The data underlying the results presented in the study are available*	All relevant data are within the manuscript and its Supporting Information files.

from (include the name of the third party and contact information or URL).<br>• This text is appropriate if the data are owned by a third party and authors do not have permission to share the data.<br><br>* typeset	
Additional data availability information:	

Powered by Editorial Manager® and ProduXion Manager® from Aries Systems Corporation

Manuscript

Click here to access/download;Manuscript;FINAL EMTs Occupational Injuries Manuscript.docx

Occupational injuries among Emergency Medical Technicians: Burden and determinants in Northern Ghana

Authors; Ali Baba Awini[1], Douglas Aninng Opoku*[2,3], Nana Kwame Ayisi-Boateng[4], Joseph Osarfo[5], Alhassan Sulemana[6], Isaac Kofi Yankson[7], Maxwell Osei-Ampofo[4], Ahmed Nuhu Zackaria[8], and Sam Newton[9]

Affiliations;

[1]Ghana National Ambulance Service, Upper East Region, Ghana.

[2]Department of Occupational and Environmental Health, School of Public Health, Kwame Nkrumah University of Science and Technology, Kumasi, Ghana

[3]Allen Clinic, Family Healthcare Services, Kumasi, Ghana

[4]Department of Medicine, School of Medicine and Dentistry, Kwame Nkrumah University of Science and Technology, Kumasi, Ghana

[5]Department of Community Medicine, School of Medicine, University of Health and Allied Health Science, Ho, Ghana

[6]Department of Environmental Science, College of Science, Kwame Nkrumah University of Science and Technology, Kumasi, Ghana

[7]Council for Scientific and Industrial Research-Building and Road Research Institute, Kumasi, Ghana

[8]Ghana National Ambulance Service, Headquarters, Accra Ghana.

[9]Department of Global and International Health, School of Public Health, Kwame Nkrumah University of Science and Technology, Kumasi, Ghana

*Corresponding author: Douglas Aninng Opoku, email: douglasopokuaninng@gmail.com

Abstract

Background: Emergency Medical Technicians (EMTs) are the main actors in the provision of prehospital emergency medical services. The operations of EMTs increase their risks of being exposed to occupational injuries. However, there is a paucity of data on the burden of occupational injuries among EMTs in sub-Saharan Africa This study, therefore, sought to estimate the burden and determinants of occupational injuries among EMTs in the Northern part of Ghana.

Methods: A cross-sectional study was conducted among 154 randomly recruited EMTs in the Northern part of Ghana. A pre-tested structured questionnaire was used to collect data on participants' demographic characteristics, facility-related factors, personal protective equipment use, and occupational injury. Multivariate logistic regression analysis with a stepwise approach was used to examine the determinants of occupational injury among EMTs.

Results: Over a period of 12 months, the prevalence of occupational injuries among EMTs was 38.6% with an overall 12-month incidence rate of 386 injuries per 1000 workers. Bruises (51.8%), and sprains/strains (14.3%) were the major types of injuries reported among the EMTs. The key determinants of occupational injury among EMTs were an absence of a health and safety committee at the workplace (aOR: 3.92, 95%CI: 1.63 – 9.43), absence of health and safety policy at the workplace (aOR: 2.76, 95%CI: 1.26 – 6.04) and dissatisfaction with health and safety measures at the workplace (aOR: 2.51, 95%CI: 1.10 – 5.71). Female EMTs had a 70% (aOR:0.30, 95%CI: 0.12 – 0.71) protection of sustaining occupational injury compared to the male EMTs.

Conclusion: Approximately three out of ten EMTs reported an occupational injury within a twelve-month period. Potential strategies to reduce the incidence of occupational injuries include the establishment of health and safety committees, formulation of health and safety policies as well as improving current health and safety measures among EMTs.

Keywords: Determinants of occupational injuries; Emergency Medical Technicians; Health and Safety measures; Ghana; Occupational injuries burden

Introduction

Emergency Medical Technicians (EMTs) are prehospital experts who take care of acutely ill or injured patients and transport them timeously to and from health facilities. EMTs assess and oversee patients' medical treatment outside the hospital setting [1–4]. They contribute significantly to a nation's healthcare continuum of care by responding to calls concerning individual patients, major disasters and mass casualty incidents. The nature of their occupation exposes them to hazards, such as road traffic accidents en route to a scene, extreme temperatures, infectious agents, noise and long working hours which can increase their risks of occupational injuries and accidents. These can include travelling in light-and-siren-filled situations, exposure to violence, shift works, lifting heavy equipment and handling patients [1,5]. It has been reported that exposure to these conditions increases the risk of EMTs to occupational injury compared to the entire population [1,6].

In the United States, data released by the National Institute for Occupational Safety and Health showed that approximately 75,400 emergency medical service workers sought care for non-fatal occupational injuries at emergency departments between 2017 and 2020 [7]. Similarly, out of a total of 21,749 cases of occupational injuries that were recorded in the USA among EMTs, 99.7% [21,690] of them resulted in non-fatality which accounted for lost work days [8]. Studies in the US and Australia have reported musculoskeletal injuries such as sprains and strains resulting from manual lifting and moving of patients as the commonest occupational injury among emergency medical service workers [4,9,10]. Occupational claims in Australia between 2003 and 2012 have been reported to be between 4.8 to 6.3 times higher among ambulance officers compared to other occupations [11]. Motor vehicle accidents, needle stick injuries, and sharp injuries have been reported by Yilmaz et al in a study as the commonest mechanism of occupational injuries among EMTs in western Turkey [12]. In South Africa, Mcdowall and Laher in a study reported that over 26.3% of emergency medical service personnel sustained needle stick injuries [13].

Factors such as temporal employment, male gender, old age, long working hours, extreme temperatures, high-speed driving, lack of personal protective equipment and poor safety culture have been reported to be associated with occupational injuries [14–19]. A study in the USA by Benavides et al reported that temporal workers had about two times increased risk of fatal injuries compared to permanent workers [17]. Another study in the USA by Reichard et al also reported that EMTs that were 45 years and above had about two times increased risk of occupational-related fatality compared to those who were 24 years and below [20]. One

possible factor that can increase the risk of occupational injury is poor adherence to personal protective equipment (PPE) use at the workplace [21]. The use of PPE (such as safety boots, reflective jackets or vests, helmets, goggles, nose masks and hand gloves) can offer protection to workers against any work-related injuries, accidents and illness.

Over the past decade, the Ghana National Ambulance Service has undergone a massive transformation since its establishment in 2004 in Ghana. Consequently, EMTS have also received standardized training and updates over this period. They receive training on basic and advanced prehospital emergency care based on their scope of practice [22]. In Ghana, prehospital personnel can offer basic, advanced or critical emergency medical services [23]. Some studies in Ghana have assessed occupational injuries [24,25], and burnout [26,27] among healthcare workers (such as nurses, physicians and laboratory technicians). However, there is no available data on the burden and determinants of occupational injury among EMTs of the Ghana National Ambulance Service. Data is required to determine the most important and cost-effective measures that could be made to enhance the health and safety of EMTs of the Ghana National Ambulance Service. This study, therefore, sought to estimate the burden and determinants of occupational injury among EMTs of the Ghana National Ambulance Service. It provides baseline data on the overall burden and determinants of occupational injuries among EMTs, which can inform policy direction to enhance the health and safety of EMTs in the Ghana National Ambulance Service. Also, it can serve as reference data in the monitoring and evaluation of interventions that may be rolled out to address injuries in this population.

Methods

Study design and setting

A cross-sectional study was conducted among EMTs of the Ghana National Ambulance Service in the then Upper East, Upper West and Northern Regions of Ghana from 30th October 2020 to 1st December 2020.

In 2008, the Ghana National Ambulance Service began providing emergency medical care (including transporting patients to and from health facilities) in these regions from just one station each, which was situated in Bolgatanga, Wa, and Tamale. The Ghana National Ambulance Service now has stations in all the regions in the northern part of Ghana, as well as a regional dispatch centre and regional administration.

The EMTs of the Ghana National Ambulance Service provide services such as the provision of pre-hospital emergency medical care to accident victims by transporting them from the road traffic crash scenes to a health facility, standby emergency cover at mass public gatherings and also liaise with other emergency care providers in time of disaster or mass casualties to provide pre-hospital care to victims. The 2022 annual report of the Ghana National Ambulance Service from the Upper East, Upper West and Northern Regions of Ghana showed that the three regions conducted 5,797 cases of hospital referrals run with an average monthly number of 483 cases in 2022. In total, there are forty-six ambulances in the three regions (Upper East: 16, Upper West: 12 and Northern Region: 18).

Study population and eligibility criteria

The study population were all EMTs of the Ghana National Ambulance Service in the three northern regions of Ghana. In total, there were two hundred and fifty-three (253) EMTs of the Ghana National Ambulance Service in the three regions (Upper East: 89, Upper West: 69 and Northern region: 95). All EMTs who have had at least twelve months of working experience with the Ghana National Ambulance Service were included in the study. All EMTs of the Ghana National Ambulance Service that were on their annual leave at the time of the study were excluded.

Sample size estimation and sampling

The sample size for this study was estimated using the Krjcie and Morgan formula for sample size calculation ($S = X^2 \text{ NP } (1 - P) \div d^2 (N - 1) + X^2 \text{ P } (1 - P)$) [28]. Where: '$X^2$' is the chi-square degree of freedom value in the table at a 95% confidence level (3.841), 'N' is the size of the population (253), P is the population proportion (29.7% of the proportion of occupational injuries sustained among health workers in Ghana [25]) and 'd' is the degree of accuracy expressed as a proportion (0.05). Using a non-response rate of 10.0%, a total of 156 participants were recruited for the study. The prevalence of occupational injuries among healthcare workers in Ghana [25] was used for the sample size estimation because there is no previous study on the injury rate among EMTs in Ghana. Also, EMTs are part of the healthcare workers in Ghana. Again, the dynamics and work environment in which EMTs operate in developed countries are very different from Ghana. This explains why a 'P' from a developed country was not used.

Proportional allocation of the sample size to the size of the target population was used to select the number of EMTs from each region. In total, 55, 43 and 58 EMTs were selected from Upper East, Upper West, and Northern regions respectively. The staff list in each region was retrieved from all the regional administration offices. Staff were assigned codes to identify them and this was used for balloting by an independent person to select the participants for each region. The EMTs that were selected were contacted on phone and scheduled appointments for the questionnaire administration.

Data collection instrument and process

A pre-tested structured questionnaire was used to collect demographic factors (age, sex, years of work, relationship status, level of education), facility-related factors (availability of health and safety committee and policy, health and safety training, provision of PPE) and occupational injury (types, contributory factors, location of accidents). The questionnaire was pretested among fifteen EMT officers in a region which was not part of the target population. The main outcome of this study was a self-reported occupational injury sustained in the last twelve months before the data collection. Occupational injury in this study was defined as any injury or accident that occurred through the performance of duty at the workplace [29]. The data was gathered using a self-administered approach among study participants. In each region, two research assistants were trained by the investigators on ethics in research and obtaining consent from research participants. The research assistants contacted the EMTs on phone to schedule an appointment for the questionnaire administration. For participants that had sustained more than one occupational injury in the last twelve months, data on their most recent injury was captured.

Data management and statistical analysis

Data entry was done in Epi Info version 7.2.4.0 (Centres for Disease Control and Prevention [CDC], USA). The data was cleaned and checked for correctness and consistency before analysis. Analysis was performed using Stata version 16 (StataCorp, College Station, USA) statistical software package. Means or medians and standard deviation or interquartile range were used to describe continuous variables while frequencies and percentages were used to describe categorical variables. Univariate and multivariate logistic regression using a backward stepwise approach was used to examine the independent determinants of occupational injury. Crude and adjusted odds ratios were presented with 95% confidence intervals. Statistical

significance was maintained at a p-value <0.05. The primary outcome of this study was the occurrence of occupational injury in the last twelve months prior to the data collection.

Ethical consideration

Ethical approval was granted by the Committee on Human Research, Publications and Ethics, School of Medicine and Dentistry, Kwame Nkrumah University of Science and Technology, Kumasi, Ghana with reference number CHRPE/AP/403/20. Study participants were briefed about the study objectives, risks and benefits of participation. They were encouraged to ask questions for clarification. All study participants signed informed consent before recruitment.

Results

Demographic characteristics of study participants

Of 156 EMTs that were recruited for the study, 154 of them completed the questionnaire with a response rate of 98.7%. The mean age of study participants was 35.5 years (±7.1) with a range of 20 to 53 years. Over 72.7% of the 154 study participants were males and over two-thirds (64.9%) had secondary education. The median years of work was 5 years (6) ranging from 1 to 21 years. Approximately 76.0% of the study participants were in the operations department (Table 1).

Table 1: Demographic characteristics of study participants

Variables	Frequency	Percentage, % [Range]
**Age group (years)**		
20 – 29	34	22.1
30 – 39	79	51.3
≥ 40	41	26.6
Mean (±SD)	35.5 (±7.1)	[20 – 53]
**Sex**		
Male	112	72.7
Female	42	27.3
**Level of education**		
Basic	5	3.3
Secondary	100	64.9
Tertiary	49	31.8
**Relationship status**		
Single	59	38.3
Married	95	61.7
**Years of work (years)**		
1 – 5	95	61.7
6 – 10	40	26.0

≥ 11	19	12.3
Median years of work (IQR)	5 (6)	[1 – 21]
**Department**		
Administration	12	7.7
Dispatch/control	25	16.3
Operations	117	76.0

IQR: Interquartile range SD: Standard deviation

Occupational injuries and personal protective equipment usage among study participants

Over a 12-month period, the prevalence of occupational injuries among the EMTs was 38.6% with an overall 12-month incidence rate of 386 injuries per 1000 workers. Out of the 59 injured, over half (51.8%) were bruises and about 39.7% of the injuries occurred at a road traffic accident scene. Over half (56.9%) of those injured reported a lack of adequate PPE as a factor that contributed to its occurrence. Approximately 70.1% of the EMTs reported that the PPE supplied to them was adequate. About two-thirds (65.6%) of the study EMTs reported that they were satisfied with the health and safety measures at their workplace. Again, over 71.4% and 52.0% of the EMTs indicated the presence of health and safety committees and policies at their workplace respectively (Table 2).

Table 2: Occupational injury, type, causes and PPE use among study participants

Variables	Frequency	Percentage, %
**Experienced injury in the past 12 months**		
Yes	59	38.6
No	94	61.4
**Type of injury (n = 56)**		
Fracture	6	10.7
Bruises	29	51.8
Sprain & strain	8	14.3
Laceration/burns	6	10.7
Others*	7	12.5
**Location of the accident (n = 58)**		
Road traffic accident scene	23	39.7
Hospital	8	13.8
Office	8	13.8
Residence of a patient	2	3.5
In the ambulance	17	29.3
**Factors affecting injuries occurrence (n = 58)**		
Inadequate training in health and safety	5	8.6
Inadequate PPE	33	56.9
Lack of knowledge of health and safety issues by the employee	11	19.0

Not sure	9	15.5
**Report injury/accident (n = 59)**		
Yes	31	52.5
No	28	47.5
**Provision of PPE**		
Adequate	108	70.1
Inadequate	46	29.9
**Usage of PPE at the workplace**		
Sometimes	49	31.8
Always	105	68.2
**Health and safety committee at the workplace**		
Yes	110	71.4
No	44	28.6
**Health and safety policy at the workplace**		
Yes	80	52.0
No	74	48.0
**Satisfaction with health and safety measures at the workplace**		
Satisfied	101	65.6
Not satisfied	53	34.4

PPE: Personal protective equipment

*Includes needle stick injuries, injuries from electrical shock, dislocation

Determinants of occupational injuries among study participants

After adjusting for significant covariates in the stepwise logistic regression, an absence of a health and safety committee at the workplace (aOR: 3.92, 95%CI: 1.63 – 9.43), absence of health and safety policy at the workplace (aOR: 2.76, 95%CI: 1.26 – 6.04) and dissatisfaction with health and safety measures at the workplace (aOR: 2.51, 95%CI: 1.10 – 5.71) were associated with occupational injury among EMTs. Female EMTs had a 70% (aOR:0.30, 95%CI: 0.12 – 0.71) protection of sustaining occupational injury compared to male EMTs (Table 3).

Table 3: factors influencing occupational injury among study participants

Variable	Injury n (%)	Crude OR 95%CI	P - value	Adjusted OR 95%CI	P - value
**Age group (years)**					
20 – 29	9 (26.5)	1.00		-	-
30 – 39	32 (40.5)	1.89 (0.78 – 4.58)	0.158	-	-
≥ 40	18 (43.9)	2.17 (0.82 – 5.79)	0.121	-	-
**Sex**					
Male	50 (44.6)	1.00		1.00	

Female	9 (21.4)	0.34 (0.15 – 0.77)	0.010	0.30 (0.12 – 0.71)	0.007
**Level of education**					
Basic	3 (60.0)	1.00		-	
Secondary	33 (33.0)	0.33 (0.05 – 2.06)	0.235	-	-
Tertiary	23 (46.9)	0.59 (0.09 – 3.85)	0.581	-	-
**Relationship status**					
Single	21 (35.6)	1.00		-	-
Married	38 (40.0)	1.21 (0.62 – 2.36)	0.585	-	-
**Years of work (years)**					
1 – 5	28 (29.5)	1.00		-	-
6 – 10	19 (47.5)	2.16 (1.01 – 4.64)	0.047	-	-
≥ 11	12 (63.2)	4.16 (1.46 – 11.50)	0.007	-	-
**Department**					
Administration	6 (50.0)	1.00		-	-
Dispatch/control	6 (24.0)	0.32 (0.07 – 1.36)	0.121	-	-
Operations	47 (40.2)	0.67 (0.20 – 2.21)	0.512	-	-
**Health and safety committee at the workplace**					
Yes	38 (34.6)	1.00		1.00	
No	21 (47.7)	1.73 (0.85 – 3.52)	0.130	3.92 (1.63 – 9.43)	0.002
**Health and safety policy at the workplace**					
Yes	23 (28.8)	1.00		1.00	
No	36 (48.7)	2.35 (1.21 – 4.57)	0.012	2.76 (1.26 – 6.04)	0.011
**Provision of PPE**					
Adequate	41 (38.0)	1.00		-	-
Inadequate	18 (39.1)	1.05 (0.52 – 2.13)	0.892	-	-
**Usage of PPE at the workplace**					
Sometimes	20 (40.8)	1.00		-	-
Always	39 (37.1)	0.86 (0.43 – 1.71)	0.662	-	-
**Satisfaction with health and safety measures at the workplace**					
Satisfied	32 (31.7)	1.00		1.00	
Not satisfied	27 (50.9)	2.24 (1.13 – 4.43)	0.021	2.51 (1.10 – 5.71)	0.029

OR: odds ratio

Discussion

The 12-month prevalence of occupational injury among study participants was 38.6% with an overall 12-month incidence rate of 386 injuries per 1000 workers. The key determinants of

occupational injury among study participants were female sex, absence of a health and safety committee at the workplace, absence of health and safety policy at the workplace and dissatisfaction with health and safety at the workplace. To our knowledge, this is the first study to estimate the burden of occupational injuries among EMTs of the Ghana National Ambulance Service.

The 36.8% prevalence rate observed in this study underpins an expanding body of evidence that EMTs are at an increased risk of occupational injuries [4,8,30,31]. The nature of their work involves bending, carrying and lifting patients [32–34] which can increase their risks of sustaining injuries. A study among 246 healthcare workers in Ghana reported a 12-month occupational injury prevalence of 29.7% [25]. Also, the injury rate (36.8%) observed in this study is similar to the prevalence of occupational injury (38.8%) reported among paramedics in a study in South Africa [19]. The commonest type of occupational injury (bruises, sprains and strains) observed among EMTs in the present study is similar to what was reported in studies among EMTs in the US [8,35].

The findings of the present study revealed that lack of adequate PPE (56.9%) was a major contributory factor to the occurrence of occupational injuries. The significance of PPE use in injury prevention has been discussed in the literature [36,37]. The health and safety of EMTs depend on high adherence to PPE use which can make the difference between safety and injuries at the workplace. The use of PPE can reduce the level of exposure to hazards when both engineering and administrative controls are not practical or efficient to minimize the risk of exposure to the barest minimum. In California, a study by Mathews et al reported that one of the barriers that hinder adherence to PPE use among paramedics is inadequate access to safety devices [38]. This study highlights the need to ensure there is an adequate supply of PPE for use to reduce the incidence of occupational injuries among EMTs in Ghana.

We observed in the present study that female EMTs had 70.0% (aOR:0.30, 95%CI: 0.12 – 0.71) protection from sustaining occupational injury compared to their male counterparts. A reason may be that females are often involved in less demanding tasks [such as administrative, and dispatch/control] which reduces their exposure risks and subsequent occurrence of occupational injuries, unlike males whose tasks may demand the use of sophisticated machines that can predispose them to high risks of injuries. This is consistent with reports in the US by the Bureau of Labour Statistics that women suffer fewer occupational injuries than men [39].

Health and safety committees work closely with management and employees in identifying potential workplace hazards and prevent them from causing accidents, illnesses and injuries at the workplace [40]. It was observed in the present study that in the absence of a health and safety committee at the workplace, EMTs had about three times (aOR: 3.92, 95%CI: 1.63 – 9.43) increased risks of sustaining occupational injury compared to those that had it at their workplace. This is consistent with reports in Canada and the USA that health and safety committee is associated with lower injury rates at the workplace [41,42]. The absence of a health and safety committee at the workplace can affect an organization's ability to routinely conduct a risk assessment which is important for identifying potential sources of hazards. Health and safety committees provide supervisory support to workers which can help to mitigate injury rates. Inadequate supervisory support has been reported by Yanar et al in a study in Canada to be significantly associated with an increased risk of physical injuries at the workplace [43].

It was observed in this study that EMTs that did not have a health and safety policy at the workplace had about two times (aOR: 2.76, 95%CI: 1.26 – 6.04) increased odds of sustaining occupational injury compared to those that had it. A possible reason could be that those without a health and safety policy may not have a good safety culture [like the use of PPE, and safety training at the workplace] and are also not likely to have a health and safety committee which is a significant strategy for injury prevention [21,44]. The existence of a health and safety policy indicates an institution's willingness to promote employee health and safety. Thus, it is in every organization's best interest to have and implement health and safety policies and measures to reduce workplace illnesses, accidents and injuries. In the United Kingdom (The Health Safety Work Act 1974 and the Management of the Health and Safety at Work Regulations 1999), it is mandatory for companies to provide health and safety policies for their staff [45,46] unlike Ghana where there is no such law.

A significant finding of this study is the two times (aOR: 2.51, 95%CI: 1.10 – 5.71) increased odds of occupational injury among EMTS that were dissatisfied with health and safety measures at the workplace compared with those that were satisfied. This may be explained by the fact that their dissatisfaction with health and safety may negatively affect their commitment to adhering to safety procedures and measures at the workplace which can increase their risk of sustaining an occupational injury. When workers perceive health and safety systems to be positive at the workplace, their level of adherence to safety regulations and measures increases [47].

Strengths and limitations of the study

This is the first study to estimate the burden and determinants of occupational injuries among EMTs in Ghana and provides useful data for future studies. A strength of this study is the representativeness of the study population in the Northern part of Ghana which enhances the generalizability of study findings. EMTs were selected from all the regional branches of the Ghana National Ambulance Service in the Northern part of Ghana. This study provides useful data for strategies to improve the health and safety of EMTs of the Ghana National Ambulance Service.

The study has some limitations. First, the possible introduction of recall bias due to the assessment of occupational injuries in the last twelve months prior to the study. There is the possibility that there could be an underestimation of the burden of occupational injuries among EMTs. Secondly, the use of cross-sectional design limits our ability to examine the causes of occupational injuries among EMTs.

Conclusion

The burden of occupational injuries was high, with 38.6% of EMTs of the Ghana National Ambulance Service reporting an injury within a 12-month period. The commonest occupational injuries sustained were bruises, sprains and strains. The occurrence of occupational injuries in this study was influenced by factors such as an absence of a health and safety committee, the absence of health and safety policies and dissatisfaction with health and safety measures at the workplace. Female EMTs were protective against occupational injuries among EMTs. We recommend the establishment of health and safety committees, formulation of health and safety policies as well as improvement of current health and safety measures (for example provision of adequate PPE and continual training on health and safety) as potential strategies to reduce the incidence of occupational injuries among EMTs.

Acknowledgement

We are grateful to the EMTs, management and staff of the Ghana National Ambulance Service in the Northern part of Ghana. We also express our profound gratitude to all the research assistants who assisted with the recruitment of study participants.

Author Contributions

Conceptualization: ABA, DAO & SN

Data curation: DAO, ABA, JO, NKAB, ANZ & IKY

Formal analysis: DAO

Methodology: ABA, DAO, NKAB, IKY, JO, AS, MOA, & SN

Project administration: MOA, ABA, NKAB, JO, AS, ANZ & SN

Supervision: SN

Validation: DAO, ABA, MOA, IKY, AS, ANZ, JO & SN

Writing – original draft: DAO, NKAB, AS, JO, & MOA

Writing – review and editing: DAO, NKAB, ABA, AS, JO, IKY, MOA, ANZ & SN

Competing interest

The authors declare that no competing interest existed for this study.

Funding

None

References

1. Sterud T, Ekeberg Ø, Hem E. Health status in the ambulance services: A systematic review. BMC Health Serv Res. 2006;6(1):1–10.

2. Maguire BJ. Violence against ambulance personnel: a retrospective cohort study of national data from Safe Work Australia. Public Heal Res Pract. 2018;28(1):1–8.

3. Maguire BJ, Neill BJO. Emergency medical service personnel's risk from violence while serving the community. Am J Public Health. 2017;107(11):1770–5.

4. Reichard AA, Marsh SM, Tonozzi TR, Konda S, Gormley MA, Reichard AA, et al. Occupational injuries and exposures among Emergency Medical Services workers. Prehospital Emerg Care. 2017;21(4):420–31.

5. Weaver MD, Patterson PD, Fabio A, Moore CG, Freiberg MS, Songer TJ. An observational study of shift length, crew familiarity, and occupational injury and illness in emergency medical services workers. Occup Environ Med. 2015;72(11):798–804.

6. Roberts MH, Sim MR, Black O, Smith P. Occupational injury risk among ambulance officers and paramedics compared with other healthcare workers in Victoria, Australia: Analysis of workers' compensation claims from 2003 to 2012. Occup Environ Med. 2015;72(7):489–95.

7. The National Institute for Safety and Health. Emergency Medical Services Workers - Injury and illness data [Internet]. 2022. Available from: https://www.cdc.gov/niosh/topics/ems/data.html [Accessed January 2023]

8. Maguire BJ, Smith S. Injuries and fatalities among Emergency Medical Technicians and Paramedics in the United States. Prehosp Disaster Med. 2013;38(4):4–10.

9. Dropkin J, Moline J, Power PM KH. A qualitative study of health problems, risk factors, and prevention among emergency medical service workers. Work. 2015;52(4):935–51.

10. Maguire BJ, O'Meara PF, Brightwell RF, O'Neill BJ FG. Occupational injury risk among Australian paramedics: an analysis of national data. Med J Aust. 2014;200(8):2008–11.

11. Gray SE, Collie A. The nature and burden of occupational injury among first responder occupations: A retrospective cohort study in Australian workers. Injury. 2017;48(11):2470–7.

12. Yilmaz A, Serinken M, Dal O, Yaylac S. Work-related injuries among emergency medical technicians in western Turkey. Prehosp Disaster Med. 2016;31(5):1–4.

13. Mcdowall J, Laher AE. Cross-sectional survey on occupational needle stick injuries amongst prehospital emergency medical service personnel in Johannesburg. African J Emerg Med. 2019;9(4):197–201.

14. Ropponen A, Koskinen A, Puttonen S, Ervasti J, Kivimäki M, Oksanen T, et al. Working hours, on-call shifts, and risk of occupational injuries among hospital physicians: A case-crossover study. J Occup Health. 2022;64(1):1–6.

15. Solmaz MS, Erdem P, Bariş G, Solmaz MS. The effects of safety culture on occupational accidents: An explanatory study in container terminals of Turkey. Int J Environ Geoinformatics. 2020;7(3):356–64.

16. Kearney J, Muir C, Smith K, Kearney J. Occupational injury among paramedics: a systematic review. Inj Prev. 2022;28(2):175–84.

17. Benavides FG, Benach J, Muntaner C, Delclos GL, Catot N AM. Associations between temporary employment and occupational injury: what are the mechanisms? Occup Environ Med. 2006;63(6):416–22.

18. Dembe AE, Erickson JB, Delbos RG BS. The impact of overtime and long work hours on occupational injuries and illnesses: new evidence from the United States. Occup Environ Med. 2005;62(9):588–97.

19. Chule N. Prevalence and risk factors of occupational injuries among emergency care providers (Doctoral dissertation). 2020;(June).

20. Reichard AA, Marsh SM, Moore PH. Fatal and nonfatal injuries among emergency medical technicians and paramedics. Prehospital Emerg Care. 2011;15(4):511–7.

21. Sawada SI, Kuklane K, Wakatsuki K MH. Editorial New development of research on personal protective equipment (PPE) for occupational safety and health. Ind HeLth. 2017;55(6):471–2.

22. Martel J, Oteng R, Anne S, Fnp B, Zakariah A, Mbbs GO, et al. The development of sustainable emergency care in Ghana: physician, nursing and prehospital care training initiatives. J Emerg Med. 2014;47(4):462–8.

23. Osei-ampofo M, Oduro G, Oteng R, Zakariah A, Jacquet G, Donkor P. The evolution and current state of emergency care in Ghana. African J Emerg Med. 2013;3(2):52–8.

24. Lori JR, Associate F, Dean A, Ms AK, Alumni FNP, Oteng R. Sharps injuries among emergency department nurses in one tertiary care hospital in Ghana. Int Emerg Nurs. 2015;28:14–9.

25. Appiagyei H, Nakua EK, Donkor P MC. Occupational injuries among health care workers at a public hospital in Ghana. Pan Afr Med J. 2021;39(1).

26. Opoku DA, Ayisi-boateng NK, Osarfo J, Sulemana A, Mohammed A, Spangenberg K, et al. Attrition of nursing professionals in Ghana : An effect of burnout on intention to quit. Nurs Res Pract. 2022;2022.

27. Opoku DA, Ayisi- NK, Mohammed A, Sulemana A, Owusuwaa A, Dominic G, et al. Determinants of burnout among nurses and midwives at a tertiary hospital in Ghana : A cross-sectional study. Nurs Open. 2022.

28. Krejcie RV MD. Determining sample size for research activities. Educ Psychol Meas. 1970;30(3):607–17.

29. International Labour Organization. Occupational safety and health statistics - ILOSTAT Database [Internet]. Available from: https://ilostat.ilo.org/resources/concepts-and-definitions/description-occupational-safety-and-health-statistics/#:~:text=An occupational injury is defined,factors arising from work activity. [Accessed December 2022]

30. Friedenberg R, Kalichman L, Ezra D, Wacht O. Work-related musculoskeletal disorders and injuries among emergency medical technicians and paramedics: A comprehensive narrative review. Arch Environ Occup Health. 2020;0(0):1–9.

31. Gülen B, Serinken M, Hatipoğlu C, Özaşır D, Sönmez E, Kaya G, et al. Work-related injuries sustained by emergency medical technicians and paramedics in Turkey. 2016;22(2):145–9.

32. Hansen CD, Rasmussen K, Kyed M, Nielsen KJ, Andersen JH. Physical and psychosocial work environment factors and their association with health outcomes in Danish ambulance personnel – a cross-sectional study. BMC Public Health. 2012;12(1):1.

33. Asa UA, Ergkvist MBA, Ngquist KÄ, Rulin CB. Relationships between work- related factors and disorders in the neck- shoulder and low- back region among female and male ambulance personnel. J Occup Health. 2005;47(6):481–9.

34. Okada N, Ishii N, Nakata M NS. Occupational stress among Japanese emergency medical technicians: Hyogo Prefecture. Prehosp Disaster Med. 2005;20(2):115–21.

35. Reichard AA. Occupational injuries among Emergency Responders. Am J Ind Med. 2010;53(1):1–11.

36. Jarl T. Fatal occupational accidents in Sweden: Personal safety equipment and rescuing. Accid Anal Prev. 1986;12(3):185–8.

37. Bull N, Riise T, Moen BE. Work-related injuries and occupational health and safety factors in smaller enterprises — a prospective study. Occup Med (Chic Ill). 2002;52(2):70–4.

38. Mathews R, Leiss JK, Lyden JT, Sousa S, Ratcliffe JM. Provision and use of personal

protective equipment and safety devices in the National Study to Prevent Blood Exposure in Paramedics. Am J Infect Control. 2008;36(10):743–9.

39. U.S Bureau of Labour Statistics. “Injuries, illnesses, and fatalities 2019.” US Dep Labour. 2020;

40. Eaton AE, Nocerino T. The effectiveness of health and safety committees: Results of a survey of public- sector workplaces the effectiveness of health and safety committees. Ind Relations A J Econ Soc. 2000;39(2):265–90.

41. Geldart S, Smith CA, Shannon HS, Lohfeld L. Organizational practices and workplace health and safety: A cross-sectional study in manufacturing companies. Saf Sci. 2010;48(5):562–9.

42. Morse T, Bracker A, Warren N, Goyzueta J, Cook M. Characteristics of effective health and safety committees: Survey results. Am J Ind Med. 2012;56(2):163–79.

43. Yanar B, Lay M, Smith PM. The interplay between supervisor safety support and occupational health and safety vulnerability on work injury. Saf Health Work. 2019;10(2):172–9.

44. Dong X, Entzel P, Men Y, Chowdhury R SS. Effects of safety and health training on work-related injury among construction laborers. J Occup Environ Med. 2004;1:1222–8.

45. The Management of Health and Safety at Work Regulations 1999 [Internet]. 1999. Available from: https://www.legislation.gov.uk/uksi/1999/3242/contents/made [Accessed December 2022]

46. Health and Safety at Work etc. Act 1974 [Internet]. 1974. Available from: https://www.legislation.gov.uk/ukpga/1974/37/contents [Accessed December 2022]

47. Amponsah-Tawaih K, Adu MA. Work pressure and safety behaviors among health workers in Ghana: the moderating role of management commitment to safety. Safety and Health at Work. 2016;7(4):340–6.

Click here to access/download

Supporting Information

DATASETS_EMTs INJURIES.dta
