# Supplementary material for: Prevalence and determinants of occupational injuries among emergency medical technicians in Northern Ghana
Source: PLoS One. 2023 Apr 25;18(4):e0284943. doi: 10.1371/journal.pone.0284943 (PMC10129006; doi:10.1371/journal.pone.0284943)
Supplement: Attachment — Submitted filename: RESPONSE TO REVIEWERS.pdf [file pone.0284943.s004.pdf]

ALLEN CLINIC
FAMILY HEALTHCARE SERVICES
P. O. Box X543 FNT, Kumasi, Ghana
Tel: 0322493429 | 0507171650
Loc: Ahensan Estates near KNUST Gate, Kumasi
Email: allenfhs@gmail.com

16th March 2023

The Editor-In-Chief
PLOS One

RESPONSE TO REVIEWERS' COMMENTS - OCCUPATIONAL INJURIES AMONG EMERGENCY MEDICAL TECHNICIANS: BURDEN AND DETERMINANTS IN NORTHERN GHANA *(MANUSCRIPT ID.* PONE-D-23-04075*).*

**Page/ Line Number**	**Components**	**Reviewers' Comments and Authors' Corrections/Responses**
		**Academic Editor**
Page 7/Lines 198-202	General comment	**Academic Editor's comments:** Both reviewers have raised these concerns in their comments on the methodology section. Please explain in clearer terms how you measured both prevalence and incidence (you stated the prevalence and incidence). I recommend you also address the other issues raised by both reviews. The manuscript will benefit from copy-editing.<br><br>**Authors' response; Thank you very much for this.** All concerns that were raised by the reviewers have been addressed. **We did not consider incidence and this has been expunged from the manuscript.** The revised section now reads as;<br><br>*…..The primary outcome of this study was the prevalence of occupational injuries in the twelve months preceding the data collection. Prevalence of occupational injuries was calculated as the proportion of EMTs that reported sustaining an occupational injury in the twelve months preceding the data collection out of all EMTs enrolled into the study and expressed as a percentage*
		**Reviewer 1**
	Title	**Reviewer's comments;** It is coherent and brief but, Northern Ghana is large areas, please specific the exact area/s<br><br>**Authors' response;** We are grateful to the reviewer for his comment on the study title. However, we respectfully would like to maintain the current study

		title. This is because this study was conducted in three out of five regions in the northern part of Ghana. In our title, 'Northern' is being used to denote the geographical area of Ghana and not the political or administrative area. We have clarified it in the study setting in the methods
Page 2/Line 37<br><br>Pages 2/Lines 47	Abstract	**Reviewer's comments;** Methods: It is well constructed, but what about why didn't include the binary logistic regression analysis in method section?<br><br>**Authors' response;** This has been done as suggested by the reviewer. The section in reference is revised to read as;<br><br>*.....Binary and multivariate logistic regression analysis with a backward stepwise approach was used to examine the determinants of occupational injury among EMTs.*<br><br>**Reviewer's comments;** Result: (aOR….) correct through your document including abstract as (AOR….)<br><br>**Authors' response;** All 'aOR' in the manuscript have been changed to 'AOR' as suggested by the reviewer.<br><br>**Reviewer's comments;** Conclusion: do not start with statement "Approximately three out of ten EMTs reported an occupational injury within a 49 twelve-month period". Please revise the conclusion part<br><br>**Authors' response; This has been revised as suggested.** The section in reference is revised to read as;<br>*….The burden of occupational injuries among EMTs of the Ghana National Ambulance Service within a 12-month period prior to data collection in this study was high*
	Introduction	**Reviewer's comments;** Well organized<br><br>**Authors' response;** Thank you
	Statements of the Problems	**Reviewer's comments;** Well organized<br><br>**Authors' response;** Thank you
	Objective	**Reviewer's comments;** Well convey<br><br>**Authors' response;** Thank you
	Methods	**Reviewer's comments; Setting:** Inconsistency usage of study area; i.e. in your title and objective, you used it Northern Ghana, but in your methods, it said (Upper East: 89, Upper West: 69 and 136 Northern regions: 95).<br><br>**Authors' response;** Upper East, Upper West and Northern regions are three out of five regions in Ghana. These regions have been created for administrative or political purposes. The study was conducted in three (Upper East, Upper West

Page 4/Lines 115-117<br><br>Page 6/Line 157<br><br>Page 5/Line 142<br><br>Page 6, 13/Lines 170-176, 336-337<br><br>Page 6/Line 172-174		and Northern regions) randomly selected out of the five regions in northern Ghana. This has been clarified in the revised manuscript. We use ‘northern Ghana’ in its geographical sense. The section in reference is revised to read as;<br><br>*….. A cross-sectional study was conducted among EMTs of the Ghana National Ambulance Service in three (Upper East, Upper West and Northern Regions) out of five regions in the Northern part of Ghana from 30th October 2020 to 1st December 2020.*<br><br>*…… First, three regions were selected at random from five regions in the northern part of Ghana.*<br><br>**Reviewer’s comments;** Please modified appropriately. **Sample size estimation and sampling:** Change as Sample size Determination<br><br>**Authors’ response;** This has been done as suggested. Thank you<br><br>**Reviewer’s comments;** I think you have two objectives: 1. Objective: Prevalence of Occ. Injuries Objective 2: Associated Factors of Occ. Injuries. So, did you calculate the sample size for associated factors? You should calculate it and compare with objective 1, and large objective will be your sample size<br><br>**Authors’ response;** Firstly, we used the prevalence to calculate the sample size because it was the primary outcome in the study. Secondly, the sample size used was reasonable even for the associated factors if one considers that most of the 95% CI around the Odds Ratios were not unreasonably wide. This suggests reasonable precision from a reasonable sample size.<br><br>**Reviewer’s comments; Data Quality:** To ensure your quality of data was/were done? Tools used for assessment., language clarity, reliability and validity?<br><br>**Authors’ response;** The data collection instrument was developed de novo by the authors through literature review and was not validated. However, to improve on its reliability and validity, it was pretested on 15 EMTs in the Ashanti Region. This has been acknowledged as a limitation in the revised manuscript. Again, data quality checks were performed by the authors by checking for correctness and consistency.<br><br>**Reviewer’s comments;** where is your pretested conducted? please address these<br><br>**Authors’ response;** It was conducted in the Ashanti Region. This is a densely populated region located in the central part of Ghana. This region is largely cosmopolitan with varied sociodemographic characteristics. This has been acknowledged in the manuscript.

Page 7/Lines 190-198<br><br>Page 7/Line 204		**Reviewer's comments; Data analysis**: How you got CRUDE ODD Ratio if you didn't use binary logistic regression. indicate the candidate variables/assumed to factors/ those moved to multivariate logistic regression. What was the modeled in the multivariate logistic regression analysis? 0.25 or 0.20 p-value please indicate again<br><br>**Authors' response;** This has been explained in the revised manuscript. The revised section now reads as;<br><br>*…..Univariate logistic regression analysis was used to obtain Crude Odds Ratio (COR) with statistical significance considered at p-value < 0.05. A multivariate logistic regression analysis using a backward stepwise approach (using a p of 0.05) was used to examine the independent determinants of occupational injuries. All variables including non-statistically significant independent variables in the univariate logistic regression were deemed important from previous studies (10,12,16,31). Hence, we adopted a backward stepwise approach and included all of them in the multivariate analysis. Statistical significance was maintained at a p-value < 0.05 with a 95% confidence interval for the final multivariate model.*<br><br>**Reviewer's comments;** Ethical consideration: Use in place Journal formal guideline<br><br>**Authors' response;** This has been revised to 'Ethics approval and consent to participate'
Page 10/Lines 245-249<br><br>Page 7/Line 192	Result	**Reviewer's comments;** Good, but it need to modify the titles of each table considering what? Where? Who? When? Example Table 1 Demographic characteristics of study participants, so here you only indicated **what and who**….<br><br>**Authors' response;** We appreciate the comments of the reviewer on the naming of the titles of the tables. However, the authors would like to maintain the current titles of all the tables as they briefly describe the contents of all the tables in the context of the study. This is also in line with the journal's requirements. Besides, there is a good chance of being repetitive.<br><br>**Reviewer's comments;** Table 3: Determinants of occupational injuries among study participants: use AOR rather than (aOR: 3.92, 95%CI: 1.63 – 9.43)<br><br>**Authors' response;** This has been done as suggested.<br><br>**Reviewer's comments;** If you used backward step<br><br>**Authors' response;** A backward stepwise approach was used and this has been acknowledged in the manuscript

	Discussion	**Reviewer's comments;** Good, some coherent paragraphs were used, please recheck it<br>**Authors' response;** Thank you. This has been done as suggested
Pages 14/Lines 342	Conclusion	**Reviewer's comments;** Modify, do not conclude with numerical or percentages [38.6%] [may use sever, high mild or low] prevalence of occupational problem/injuries is found among……………, which need…………………<br><br>**Authors' response;** This has been done as suggested. The revised section now reads;<br>*.....The burden of occupational injuries among the EMTs of the Ghana National Ambulance Service in this study was high*
Page 14-19	References	**Reviewer's comments;** DOI is required for those have<br><br>**Authors' response;** The DOI have been provided as recommended.
	General Comments	**Reviewer's comments;** Good research areas and article, but it need major revision.<br>**Authors' response;** Thank you for your comments. The manuscript has been revised based on reviewers' comments. We are very grateful.
		**Reviewer 2**
Page 2/Line 28<br><br>Page 2/Line 31<br><br>Page 7/Lines 198-202	Abstract	**Reviewer's comments; ….**(EMTs) are the primary providers of….<br><br>**Authors' response;** This has been done as suggested<br><br>**Reviewer's comments; …** However, there is a paucity of data on the burden of occupational injuries among EMTs in sub-Saharan Africa This study… place a 'full stop' after Africa<br><br>**Authors' response;** This has been done.<br><br>**Reviewer's comments;** You seem to be using the words prevalence and incidence interchangeably. Your methods show that you are focused on incidence.<br><br>**Authors' response;** The main outcome of the work was to estimate the prevalence of occupational injury. We have reported only prevalence in the revised manuscript. Thank you.<br><br>The revised section now reads as;<br><br>*.....The primary outcome of this study was the prevalence of occupational injuries in the twelve months preceding the data collection. Prevalence of occupational injuries was calculated as the proportion of EMTs that reported sustaining an occupational injury in the twelve months preceding the data collection out of all EMTs enrolled into the study and expressed as a percentage*

Page 2, 10/Lines 43, 245		**Reviewer's comments;** 'Protection' is an unclear word in this context. Instead, I recommend that you report the AOR for men.<br><br>**Authors' response;** This has been done as suggested. We have presented the AOR for men as suggested in the revised manuscript.
	Introduction	**Reviewer's comments;** Good introduction<br>**Authors' response;** Thank you
	Methods	**Reviewer's comments;** The main outcome of this study was a self-reported occupational injury sustained in the last twelve months before the data collection (highlighted).<br><br>**Authors' response;** This has been deleted from the data collection instrument and process section as highlighted
Page 7/Line219	Results	**Reviewer's comments; Define what 6 means in the bracket….** The median years of work was 5 years (6)<br><br>**Authors' response;** This has been defined as suggested. The revised section now reads;<br>The median years of work was 5 years (interquartile range: 6).
Page 12/Line 287<br><br>Page 12/Line 292<br><br>Page 12/Line 294<br><br>Page 12/Line 303	Discussion	**Reviewer's comments;** 'Labor' instead of 'labour'<br><br>**Authors' response;** This has been corrected as suggested<br><br>**Reviewer's comments;** EMTs had about 'four' and not 'three' times (aOR: 3.92, 95%CI: 1.63 – 275 9.43)<br><br>**Authors' response;** This has been corrected as suggested<br><br>**Reviewer's comments;** Add 'a' between that and health….. in Canada and the USA that health and safety….<br><br>**Authors' response;** This has been done as suggested.<br><br>**Reviewer's comments;** ….had about two times (aOR: 2.76, 95%CI: 1.26 – 6.04) Usually round to the closest whole number. So "three" here.<br><br>**Authors' response;** This has been done as suggested
Page 16, 19/Lines 432, 501	References	**Reviewer's comments;** Ideally add the name of the university and, if possible, a URL. Also, for this type of reference a URL is ideally included.<br><br>**Authors' response;** This has been done as suggested

We hereby submit our revised manuscript for your consideration.

Sincerely,

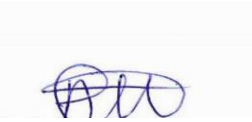

DOUGLAS ANINNG OPOKU
CORRESPONDING AUTHOR
