# Supplementary material for: Prevalence and determinants of occupational injuries among emergency medical technicians in Northern Ghana
Source: PLoS One. 2023 Apr 25;18(4):e0284943. doi: 10.1371/journal.pone.0284943 (PMC10129006; doi:10.1371/journal.pone.0284943)
Supplement: Attachment — Submitted filename: RESPONSE TO REVIEWERS.pdf [file pone.0284943.s006.pdf]

ALLEN CLINIC
FAMILY HEALTHCARE SERVICES
P. O. Box X543 FNT, Kumasi, Ghana
Tel: 0322493429 | 0507171650
Loc: Ahensan Estates near KNUST Gate, Kumasi
Email: allenfhs@gmail.com

8th April 2023

The Editor-In-Chief
PLOS One

RESPONSE TO REVIEWERS' COMMENTS – PREVALENCE AND DETERMINANTS OF OCCUPATIONAL INJURIES AMONG EMERGENCY MEDICAL TECHNICIANS IN NORTHERN GHANA *(MANUSCRIPT ID.* PONE-D-23-04075R1*).*

**Page/ Line Number**	**Components**	**Reviewers' Comments and Authors' Corrections/Responses**
		**Academic Editor**
	General comment	**Academic Editor's comments:** The reason for my decision of a major revision is because of the sample size formula you used for calculation of sample size. That formula is for small sample techniques and some of the assumptions in that formula don't reflect general opinion. One of the main purposes of sample size calculations for estimating prevalence is representativeness. Up and unless the calculated sample size is bigger than the known finite source population, there is no need to apply a constraining formula like the Krejcie formula. I request that you attempt a re-calculation with the Cochrane formula in determining the sample size for one proportion: n = (Zsq x PQ) /dsq. Since you used a sampling method other than simple random sampling, there is need to account for the dilution of variability. This is done by multiplying the above formula with a design effect value (usually>=1.5). It is not necessary to compute a design effect here so you can assume a design effect value from 1.5 and above. If after recalculation with varying assumptions, you do not get a sample size that works; the study will benefit from putting a cautionary statement in the discussion that reflects this.<br><br>**Authors' response;** We have used the Cochrane formula in determining the sample size for one proportion as suggested. We further adjusted the sample size using the finite population correction formula. This approach has been used in other studies [1-4]. The revised section on the sample size estimation now reads;<br><br>*The sample size for this study was estimated using Cochran's formula for sample size calculation:* $Sample\ size, SS = \frac{Z^2pq}{d^2}$*, where 'Z' is the chi-square degree of*

Page 10/Lines 144-160		*freedom value in the table at a 95% confidence level (1.96), 'p' is the population proportion (29.7% of the proportion of occupational injuries sustained among health workers in Ghana (25)), q is (1 – p) and 'd' is the degree of accuracy expressed as a proportion (0.05). The sample size was estimated as follows:* $Sample\ size, SS = \frac{1.96^2\ x\ 0.297\ x\ (1 - 0.297)}{0.05^2}$ $= 321 \quad Equation\ 1$ *The sample size was adjusted to 142 using the finite population correction formula for the study using Equation 2:* $New\ sample\ size, n = \frac{SS}{1 + \frac{SS - 1}{N}} \quad Equation\ 2$ *where n is the new sample size, SS is the estimated sample size, 321, and N is the finite population, 253 (which is the total number of EMTs in the three regions). Using a non-response rate of 10.0%, a total of 156 EMTs were recruited for the study. The prevalence of occupational injuries among healthcare workers in Ghana (25) was used for the sample size estimation because there is no previous study on the injury rate among EMTs in Ghana. Also, EMTs are part of the healthcare workers in Ghana. Again, the dynamics and work environment in which EMTs operate in developed countries are very different from Ghana.* <br>***References*** 1. *Agu AP, Umeokonkwo C, Adeke A, Nnabu CR, Ossai E, Azuogu B. Awareness of occupational hazards, use of personal protective equipment and workplace risk assessment among welders in mechanic village, Abakaliki, Southeast Nigeria: Awareness of occupational hazards among welders. Nigerian Medical Journal. 2021;62(3):113-21.* 2. *Bentum L, Brobbey LK, Adjei RO, Osei-Tutu P. Awareness of occupational hazards, and attitudes and practices towards the use of personal protective equipment among informal woodworkers: the case of the Sokoban Wood Village in Ghana. International Journal of Occupational Safety and Ergonomics. 2022 Jul 3;28(3):1690-8.* 3. *Mbaisi EM, Wanzala P, Omolo J. Prevalence and factors associated with percutaneous injuries and splash exposures among health-care workers in a provincial hospital, Kenya, 2010. Pan African Medical Journal. 2013 Apr 29;14(1).* 4. *Ofori AA, Osarfo J, Agbeno EK, Manu DO, Amoah E. Psychological impact of COVID-19 on health workers in Ghana: A multicentre, cross-sectional study. SAGE Open Medicine. 2021 Mar;9:20503121211000919.* <br>**Academic Editor's comments:** Your title should be changed to reflect the view of the reviewer. However, replace "burden" with "prevalence" in the title and all through the text since you did not actually deal with burden

Page 1l/Lines 1-2		**Authors' response;** This has been done as suggested. All "burden" in the manuscript have been replaced with "prevalence" The revised title now reads as;<br><br>*Prevalence and determinants of occupational injuries among Emergency Medical Technicians in Northern Ghana*
**Reviewer 1**		
Page 1l/Lines 1-2	Title	**Reviewer's comments;** I prefer it if your title will be modified as "Burden and determinants of Occupational injuries among Emergency Medical Technicians in Northern Ghana"<br>**Authors' response;** The study title has been revised based on your comment and that of the Academic Editor.<br>The revised title now reads as;<br><br>*Prevalence and determinants of occupational injuries among Emergency Medical Technicians in Northern Ghana*
Page 2/Lines 47-51	Abstract	**Reviewer's comments;** Methods: Good<br><br>**Authors' response;** Thank you<br><br>**Reviewer's comments;** Methods: good<br><br>**Authors' response;** Thank you<br><br>**Reviewer's comments;** Results: good<br><br>**Authors' response;** Thank you<br><br>**Reviewer's comments;** Still I'm not satisfied with the conclusion, hence need rewrite again. I prefer it if your title will be modified as "In the twelve months before to the data collection for this study, the burden of occupational injuries among EMTs of the Ghana National Ambulance Service was high. The creation of health and safety committees, the creation of health and safety rules, and strengthening current health and safety procedures for EMTs are all possible ways to lessen this"<br><br>**Authors' response;** This has been revised as suggested. The section in reference is revised to read as;<br><br>.....*In the twelve months before to the data collection for this study, the prevalence of occupational injuries among EMTs of the Ghana National Ambulance Service was high. The creation of health and safety committees, the creation of health and safety rules, and strengthening current health and safety procedures for EMTs are all possible ways to lessen this*.

Page 2/Lines 54-55		**Reviewer's comments;** Keywords: Should be arranged in alphabetical, it is not seeking a long phrase, it is about words, thus correct each long phrase used for MeSH, Revise it again<br><br>**Authors' response;** This has been revised as suggested.
Page 3/line 85-88	Introduction	**Reviewer's comments; Please use** risks of occupational injuries mentioned line#66 should be moved to risk factors found between line #84-#94. Minor revision of introduction<br><br>**Authors' response;** This has been done as suggested. Thank you
Page 10/Lines 144-160	Methods	**Reviewer's comments; Setting:** Source of information under study area? Line #115-131 should be cited<br><br>**Authors' response;** This has been done as suggested<br><br>**Reviewer's comments;** Sample size calculation: Use standard sample size calculation for on (S = X 2 NP (1 – P) ÷ d 2 (N – 1) + X 2 P (1 – P) among "equations" found on the task word like the following style, it is not your, but do in same fashion.<br>$S=\frac{x^2(NP(1-P)}{d^2(N-1)}+\cdots$<br><br>**Authors' response;** We have presented the sample size equation in the fashion recommended by the reviewer<br><br>**Reviewer's comments;** What does "This explains why a 'P' from a developed country was not used?" mean line 153-154<br><br>**Authors' response;** This statement has been removed from the revised manuscript
	Result	**Reviewer's comments;** I suggesting you please avoid each bold of variables (such as **Age group, sex**,… found Table 1 , **Experienced injury in the** past 12 months…etc in Table 2, associated factors (**Age, education**……mentioned in Table 3<br><br>**Authors' response;** This has been done as suggested. Thank you.
	Discussion	**Reviewer's comments; Inappropriate,** line 257-259: The key determinants………..<br><br>**Authors' response;** This has been removed from the revised manuscript.<br><br>**Reviewer's comments; unnecessarily statement,** line 259-261: To the best to……..<br>**Authors' response;** This has been removed from the revised manuscript.

Page 11-12/Lines 264-276		**Reviewer's comments;** I strongly advise you to compare and contrast the current main finding with other studies with potential discrepancies once you have mentioned the current main finding<br><br>**Authors' response;** This has been done as suggested. We could not find many prevalence studies on EMTs injuries to compare our findings. The studies that have been conducted about the subject estimated incidence of injury rate per full-time equivalence which makes it difficult for direct comparison since we estimated prevalence. The revised section now reads;<br><br>*.... Reichard et al reported an injury rate of 8.6 per 100 full-time equivalents among emergency medical service workers in the USA [4]. Maguire et al also in a retrospective analysis of data on all occupational injuries reported an injury rate of 34.6 per 100 full-time workers per year in the USA [36]. Yilmaz et al reported an incidence rate of injuries of 10.9 among EMTs in Turkey [11]. However, all these studies [4, 11, 36] reported incidence rates which makes it difficult to directly compare to the prevalence rate in the present study. Reichard et al utilized records of all occupational injuries collected through the National Electronic Injury Surveillance System from 2010 to 2014 in the USA which can account for the high incidence rate of occupational injuries in their study [4]. Similarly, the high incidence rate of occupational injuries reported by Maguire et al USA [36] could also be attributed to the use of injury records from two urban agencies' registries on all reported cases of occupational injuries from 1998 to 2002 in the USA. In Ghana, there is no such system in place that keeps records of all occupational injuries.*
Page 14/Lines 349-356	Conclusion	**Reviewer's comments;** Still I'm not satisfied with the conclusion, hence need rewrite again<br><br>**Authors' response;** The conclusion has been revised as suggested. Thank you. The section in reference now reads as;<br><br>*In the twelve months before to the data collection for this study, the prevalence of occupational injuries among EMTs of the Ghana National Ambulance Service was high. The commonest occupational injuries sustained were bruises, sprains and strains. The occurrence of occupational injuries in this study was influenced by factors such as male EMTs, an absence of a health and safety committee, the absence of health and safety policies and dissatisfaction with health and safety measures at the workplace. The creation of health and safety committees, the creation of health and safety rules, and strengthening current health and safety procedures for EMTs are all possible ways to lessen this.*

We hereby submit our revised manuscript for your consideration.

Sincerely,

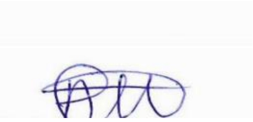

DOUGLAS ANINNG OPOKU
CORRESPONDING AUTHOR
