# Supplementary material for: Prevalence and determinants of occupational injuries among emergency medical technicians in Northern Ghana
Source: PLoS One. 2023 Apr 25;18(4):e0284943. doi: 10.1371/journal.pone.0284943 (PMC10129006; doi:10.1371/journal.pone.0284943)
Supplement: Attachment — Submitted filename: Reviewer_Feedback on PONE_D_23_04075.docx [file pone.0284943.s002.docx]

PLOS ONE: Comments and Suggestion for Authors

Occupational injuries among Emergency Medical Technicians: Burden and determinants in Northern Ghana: ID: PONE-D-23-04075

| S.no | Components | My Comments and Suggestions | Expect from Author’s Reflection |
| --- | --- | --- | --- |
| 1. | Title | - It is coherent and brief but, Northern Ghana is large areas, please specific the exact area/s |  |
| 2. | Abstract | - Methods: It is well constructed, but what about why didn’t include the binary logistic regression analysis in method section? - Result: (aOR….) correct through your document including abstract as (AOR….) - Conclusion: do not start with statement “Approximately three out of ten EMTs reported an occupational injury within a 49 twelve-month period”. Please revise the conclusion part |  |
| 3. | Introduction | Well organized |  |
| 4. | Statements of the Problems | Well organized |  |
| 5. | Objective | Well convey |  |
| 6. | Methods | - **Setting:** Inconsistency usage of study area; i.e. in your title and objective, you used it Northern Ghana, but in your methods, it said (Upper East: 89, Upper West: 69 and 136 Northern regions: 95). Please modified appropriately. - **Sample size estimation and sampling:**  1. Change as Sample size Determination 2. I think you have two objectives 3. Objective: Prevalence of Occ. Injuries 4. Objective 2: Associated Factors of Occ. Injuries . So, did you calculate the sample size for associated factors? You should calculate it and compare with objective 1, and large objective will be your sample size  - **Data Quality:** To insure your quality of data was/were done?  1. Tools used for assessment 2. Language clarity 3. Reliability and validity? 4. where is your pretested conducted? please address these  - **Data analysis**:  1. How you got CRUDE ODD Ratio if you didn’t use binary logistic regression. 2. indicate the candidate variables/assumed to factors/ those moved to multivariate logistic regression 3. What was the   modeled in the multivariate logistic regression analysis? 0.25 or 0.20 p-value please indicate again   - **Ethical consideration**: Use in place Journal formal guideline |  |
| 7. | Result | - Good, but it need to modify the titles of each table considering  1. what? 2. Where? 3. Who? 4. When? 5. Example   Table 1 Demographic characteristics of study participants, so here you only indicated **what and who**….   - Table 3: Determinants of occupational injuries among study participants: use AOR rather than (aOR: 3.92, 95%CI: 1.63 – 9.43) - If you used backward step |  |
| 8. | Discussion | - Good, some coherent paragraphs were used, please recheck it |  |
| 9. | Conclusion | - Modify, do not conclude with numerical or percentages [38.6%] - [may use sever, high mild or low] prevalence of occupational problem/injuries is found among………………, which need…………………. |  |
| 10. | References | - DOI is required for those have |  |
| 11. | General Comments | - Good research areas and article, but it need major revision. |  |
