# Supplementary material for: Prevalence and determinants of occupational injuries among emergency medical technicians in Northern Ghana
Source: PLoS One. 2023 Apr 25;18(4):e0284943. doi: 10.1371/journal.pone.0284943 (PMC10129006; doi:10.1371/journal.pone.0284943)
Supplement: Attachment — Submitted filename: Comments_PONE-D-23-04075R1.docx [file pone.0284943.s005.docx]

**Title**

I prefer it if your title will be modified as “Burden and determinants of Occupational injuries among Emergency Medical Technicians in Northern Ghana”

**Abstract**

1. *Introduction*…Good
2. *Objective*…Good
3. *Method*…Good
4. *Result* …..good
5. *Conclusion*

- Still I’m not satisfied with the conclusion, hence need rewrite again

I prefer it if your title will be modified as “In the twelve months before to the data collection for this study, the burden of occupational injuries among EMTs of the Ghana National Ambulance Service was high. The creation of health and safety committees, the creation of health and safety rules, and strengthening current health and safety procedures for EMTs are all possible ways to lessen this”

6. *Keywords*: Should be arranged in alphabetical, it is not seeking a long phrase, it is about words, thus correct each long phrase used for MeSH, Revise it again

**Introduction**

- **Please use** risks of occupational injuries mentioned line#66 should be moved to risk factors found between line #84-#94. Minor revision of introduction

**Methods**

- Source of information under study area?
- Line #115-131 should be cited
- Sample size calculation: Use standard sample size calculation for on ( S = 𝑋 2 NP (1 – P) ÷ 𝑑 2 (N – 1) + 𝑋 2 P (1 – P) among “equations” found on the task word like the following style, it is not your, but do in same fashion.

S=$\frac{x^{2}(NP(1-P)}{d^{2}(N-1)}+\ldots$

- What does “This explains why a ‘P’ from a developed country was not used?” mean line 153-154

**Result**

- I suggesting you please avoid each bold of variables (such as **Age group, sex**,… found Table 1 , **Experienced injury in the** past 12 months…etc in Table 2, associated factors (**Age, education**……mentioned in Table 3

**Discussion**

- **Inappropriate,** line 257-259: The key determinants………..
- **unnecessarily statement,** line 259-261: To the best to……..
- **I strongly advise you to compare and contrast the current main finding with other studies with potential discrepancies once you have mentioned the current main finding**

**Conclusion**

- Still I’m not satisfied with the conclusion, hence need rewrite again
